# Mediocre or excellent-where does your facility stand? Becoming a perinatal loss gold standard hospital

**DOI:** 10.1186/1471-2393-15-S1-A13

**Published:** 2015-04-15

**Authors:** Sue Steen, Sherokee Ilse

**Affiliations:** 1 Sue Steen, Maple Grove Hospital, Maple Grove, MN, USA

## 

During times of limited funding and health care challenges, hospitals and clinics must continue their commitment to give excellent, ‘gold standard’ care to bereaved families. Since families only get one chance to “do it right” when their baby dies in miscarriage, stillbirth, and infant death, they deserve excellent care. A premier perinatal loss program needs solid leadership; administrative support; financial resources; system-wide communication and coordination; and well-trained and supported staff, who can then offer individualized patient-based, comprehensive, and compassionate care to bereaved families. [1,2]

Hospital management that supports the development, continuity, and ongoing financial support of a well-planned and implemented hospital-wide perinatal loss program, is the first component found in a Gold Standard program. All areas of the hospital that work with perinatal loss, clinics, emergency departments, surgical services, and the birth center, must be included in the program. Financial support allows for staff hours dedicated for bereavement support and budget for education, training, materials, resources and events. [1]

The second component of the program is that all staff is appropriately and routinely trained, mentored, and supported, and that the program is based on comprehensive national standards, protocol, and policies [3]. Annual, mandatory education of staff, frequent updates, area workshops, and clear guidelines are essential [1,4]. Mentoring of new or unsure staff, support with paperwork, and help with memory making activities are important areas of focus. Recognition and support of staff is also essential for a successful program.

The final component of the program is that there is an integrated process that offers seamless, excellent care to each and every family from the time of their diagnosis, during their hospital stay, and with ongoing care by their medical provider team, including clinic points of contact. Initial communication and referral upon diagnosis is critical for informing and educating families regarding their options [2]. When possible, slowing hospital admittance and sharing practical resources is recommended to assist families in preparation for the birth of their baby. This strategy usually results in lessened shock and more control for the parents. Birth planning and companioning, which is presently being offered by trained hospital/clinic staff, local care companions, and by Baby Loss Advisors™, can provide families with individualized, culturally specific care [5]. This care and support occurs prior to induction, throughout the process of meeting and saying goodbye to the baby, and continues post hospital discharge. When this type of adjunct care paradigm is utilized, parents have a better chance of receiving comprehensive, consistent, and ongoing care.

Bedside care of the family includes involvement of extended family and friends, sibling care, memory making, and education of parents regarding handling of baby’s body and funeral planning [6,7]. Consistent, informed caregivers are critical during this difficult time for the family. The time of saying good-bye to the baby has been found to be one of the most difficult times during the family’s bereavement experience. Bereavement and medical discharge planning allows for a time to provide education, resources and a transition to home after the loss. Follow-up care involves supportive phone calls, support groups, and other touch points such as community memorial events [8]. Such ongoing care may help the family continue to heal.

Barriers to the Gold Standard program are related to a lack of financial resources, staffing issues and needs, healthcare provider’s lack of interest, and communication challenges. However, a designated individual, who is responsible for advocating and directing the program, can work with leaders and providers to put bereaved families first and help them honour the lives of their babies. [2].

A *Perinatal Loss Gold Standard* manual, which includes checklists, assessment tools, and policy templates, was presented as one option to aid hospitals in developing and improving their own program [9]. The RTS program was also offered as another resource for hospital program training and development [1].
